# Exploring Age-Related Variations in Carpal Bone Volume: Implications for Clinical Practice and Anatomical Understanding

**DOI:** 10.1177/15589447241242830

**Published:** 2024-04-12

**Authors:** Aparna D. Ganhewa, Ishith Seth, Rui Wu, Michael P. Chae, Vicky Tobin, Julian A. Smith, David J. Hunter-Smith, Warren M. Rozen

**Affiliations:** 1 Department of Plastic, Reconstructive and Hand Surgery, Peninsula Health, Frankston, VIC, Australia; 2 Peninsula Clinical School, Central Clinical School, Monash University, The Alfred Centre, Melbourne, VIC, Australia; 3 Department of Surgery, School of Clinical Sciences, Monash Medical Centre, Clayton, VIC, Australia

**Keywords:** carpus, wrist, aging, bones, anatomy, morphology

## Abstract

**Background:**

Clinically recognizing the changes in carpal bone volumes and understanding their implications in predicting osteoarthritis (OA) is crucial in clinical practice This study aimed to explore age-related differences in carpal bone volumes across genders, leveraging computed tomography (CT) wrist scans to create 3D surface models of these bones.

**Methods:**

Carpal bone volumes were calculated using the 3D Slicer software from CT scans obtained from Frankston Hospital and additional datasets from Brown and Auckland Universities. The data were statistically processed using Stata V13. Double-sided *P*-values < .05 were considered statistically significant. The study was conducted in accordance with the ethical standards laid out in the Declaration of Helsinki.

**Results:**

A total of 181 patients were analyzed, and 48% of whom were female. A statistically significant positive Spearman correlation (rho = 0.37-0.611, *P* <.05) was observed between increasing age and the volume of all surveyed carpal bones (scaphoid, lunate, triquetrum, pisiform, hamate, capitate, and trapezium) across genders. Intrauser and interuser reliabilities for 3D Slicer–generated volumes of trapezium and pisiform bones were statistically significant, with Interclass Correlation Coefficient (ICC) values of 0.86 and 0.95, respectively.

**Conclusion:**

Trapezial volumes increase with age, potentially due to the presence of OA and consequent osteophyte formation. This pattern is more prevalent among older individuals and women. However, the positive correlation between carpal bone volume and age was consistent across all carpal bones and both genders, regardless of OA presence. These findings suggest that carpal bone volume may naturally increase with age, independent of OA-related changes.

**Level of Evidence:**

III, cohort study.

## Introduction

The human hand is a complex structure, orchestrated by intricate relationships between bones, ligaments, tendons, and muscles to perform a multitude of tasks.^1^ The carpus, or the wrist, is a central part of this intricate structure, consisting of eight carpal bones arranged in two rows.^2^ While traditional understanding suggests that bone growth halts after reaching skeletal maturity in adulthood, ongoing research indicates that these bones undergo significant changes throughout life. Comprehending the age-related changes in carpal bones is essential for understanding their impact on the development and management of osteoarthritis (OA), enhancing the ability to predict OA risk and improve clinical management strategies.^3^

Bone continuously remodels throughout a person’s life due to the interplay between osteoblasts and osteoclasts. Most age-related literature on bone focuses on the decline in bone mineral density, leading to osteoporosis, or OA in older individuals.^3^ Beyond density changes and OA, bones also experience physiological morphological alterations across adulthood and into old age. As individuals age, their carpal bones face modifications that can compromise their function and promote OA. A significant change observed is the decrease in carpal bone volume, which is associated with an increased risk of OA.^4^ This volume loss stems from decreased bone density and increased porosity, predisposing the bones to fractures.^4^ Consequently affected bones exhibit increased stiffness, diminished strength, and a general decline in hand function quality.^5^ Advanced imaging such as magnetic resonance imaging (MRI) and computed tomography (CT) scans can detect these changes.

Clinically, recognizing alterations in carpal bone volumes and their implications for OA prediction can improve diagnostic precision, shape treatment approaches, and promote earlier intervention, ultimately enhancing patient management outcomes in OA.^5,6^ Proactive interventions including lifestyle modifications, physical therapy, or pharmacological approaches can potentially delay or mitigate the severity of OA. Moreover, a nuanced grasp of age-related changes in carpal bone anatomy can enhance surgical techniques and enable more personalized postoperative recovery plans for older patients.^3-5^ Despite the importance of this topic, there is paucity of literature concerning detailed morphological and anatomical data on age-related changes in carpal bones. Therefore, this study aims to elucidate the natural variations in carpal bone size across different age groups for both men and women.

## Materials and Methods

### Ethics Approval

A cross-sectional anatomical study of the carpal bones was conducted using in vivo anatomic data from clinical CT scans. Ethics approval was granted for the use of anonymized CT wrist scans through the Peninsula Health, and informed consent was not considered necessary as the study received low-risk ethics approval from Low-Risk Ethics Committee at Frankston Hospital Human Research Ethics Committee Hospital (PHLNR#201907). The study was conducted in accordance with the ethical standards laid out in the Declaration of Helsinki.

### CT Wrist Selection

A total of 60 CT wrist scans, featuring slice thicknesses ranging from 0.5 to 1 mm, were acquired from the Radiology Department at Frankston Hospital. All scans were anonymized to remove any identifiable information. Scans were excluded from the study if they did not capture the entire carpus, contained any metal implants, or showed signs of avascular necrosis in any of the carpal bones. For each scan, demographic data of age and gender were recorded, along with the side of the wrist scanned (left or right), the clinical reason for the CT scan, and the Eaton-Littler stage of OA at the base of the thumb, as determined through sagittal views. Scans were classified as Stage I OA if there was no observable OA at the base of the thumb.^7^

### Evaluation of Carpal Bone Size

Volumetric analysis of the carpal bones first required the CT images to be processed using a Computer Aided Design (CAD) program to create a CAD file.^8-10^ The volume of each carpal bone was derived by creating 3D surface models (SMs) using 3D Slicer (Surgical Planning Laboratory, Boston, Massachusetts) through a process of automated and manual segmentation.^11^ CT images in the DICOM format were uploaded to the 3D slicer program, version 4.6.2. Utilizing the “Editor” module, a “thresholding effect” was employed to generate a unified label map. This effect uses Hounsfield units to selectively highlight structures based on their density. The threshold settings were fine-tuned to focus specifically on the carpal bone of interest while excluding extraneous background structures.

**Figure 1.**
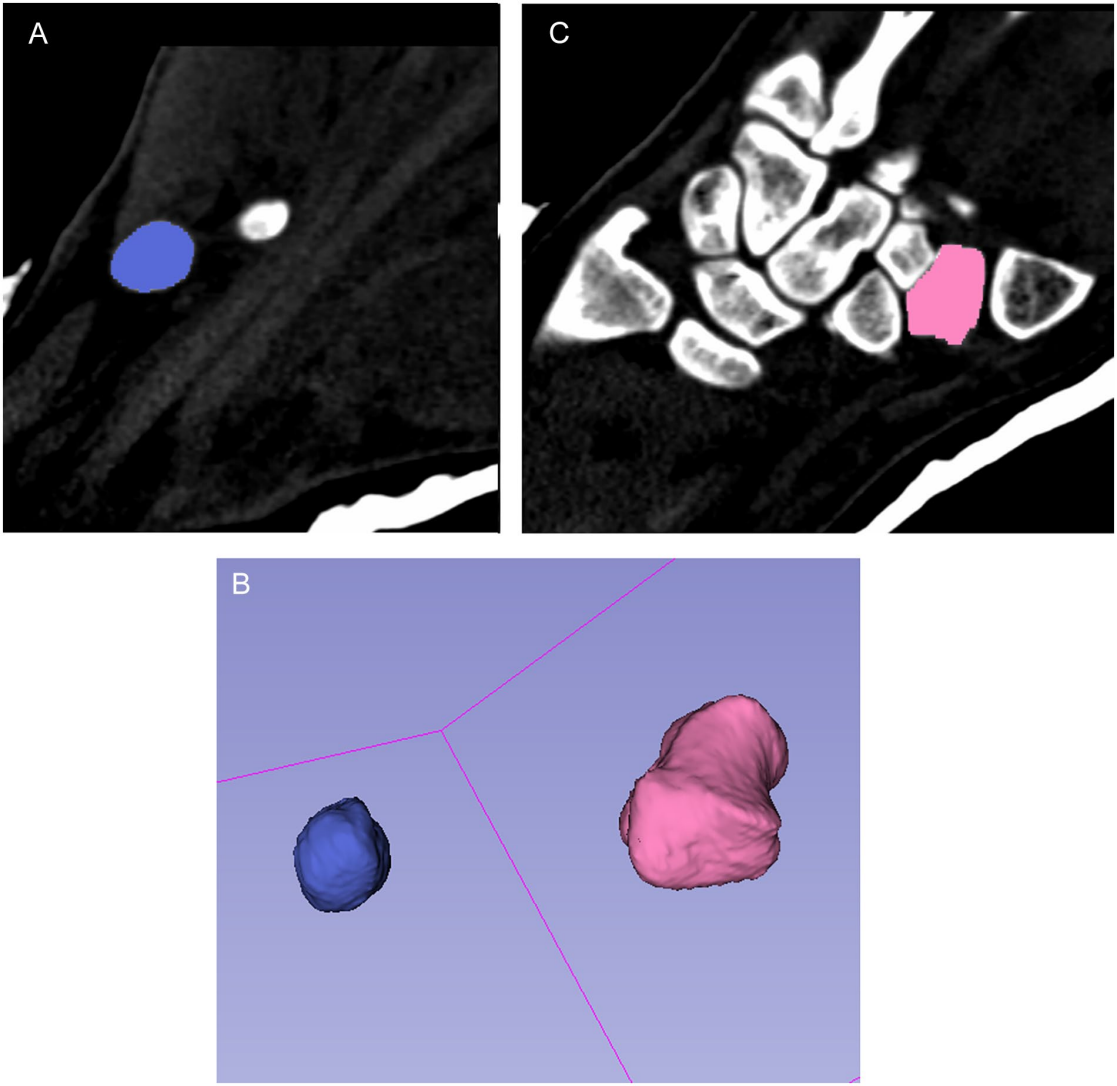
Screenshot of segmented pisiform (A) and trapezium (B) and 3D virtual surface model of trapezium and pisiform (C).

After setting the optimal threshold, any other labels connected to the targeted carpal bone were meticulously removed. This procedure was executed slice by slice across all imaging planes—coronal, sagittal, and transverse—until the carpal bone appeared completely isolated. At this point, features such as the “change island effect” or “save island effect” were activated to distinguish the carpal bone with a unique color label and to eliminate all unrelated background elements. Using the “paint effect,” the isolated carpal bone was then consistently colored across all slices and planes. Once the segmentation of the carpal bone was deemed satisfactory, a 3D SM was constructed using the “make model effect” (Figure 1). This model, along with its detailed attributes like volume, could then be inspected in the “Models” module.

### Evaluation of User Variably in 3D Slicer–Generated Carpal Bone Volume

To assess both interuser and intrauser variability in the volumetric outcomes of the carpal bone SMs, multiple models were generated from identical CT wrist scans by the same individual on 2 separate occasions, as well as by 2 different individuals. For evaluating intrauser variability, one researcher (ADG) created SMs of the trapezium and pisiform bones using 50 different CT scans. The process was then repeated after renaming the scans to blind the researcher, ensuring that the initial set of measurements did not influence the second set. For interuser variability assessment, SMs of the trapezium and pisiform bones were constructed from 50 CT scans by 2 different researchers (ADG and RW). The volume measurements from each scan, as determined by both researchers, were then compared. The Interclass Correlation Coefficient (ICC) was employed to quantify the level of reliability and agreement between the measurements taken by a single user and those taken by multiple users.

### Inclusion of Additional Datasets

Further datasets investigating carpal bone volume were obtained through collaboration with research groups from Brown University (Providence, Rhode Island) and Auckland University (Auckland, New Zealand).^12^ The main purpose of collecting additional datasets was to increase numbers in the study and to provide a more comprehensive database of carpal bone volume.

The dataset from Brown university contained the volumes of all carpal bones from 60 young (age < 30 years) and healthy individuals, without evidence of base-of-thumb OA. The findings from this dataset have been previously published, and the authors have made the data publicly available for future research.^13^ The volume measurements were extracted from CT images via segmentation and subsequent SM creation. In contrast, the dataset from Auckland University, which has also been published, was not previously accessible for collaborative research.^12^ This dataset specifically focused on the volumes of the trapezium bone from 61 individuals, all of whom showed no evidence of base-of-thumb OA. Similar to the Brown University dataset, the volumes were calculated using CT images and segmentation techniques.

### Statistical Analysis

Statistical analysis was conducted using Stata version 13. Given the recognized gender differences in carpal bone size, the data were stratified by gender to allow for more nuanced interpretation.^14^ A Spearman correlation test was employed to ascertain any significant relationships between carpal bone volume and age.

## Results

### Demographics of Peninsula Health

A total of 60 CT scans were included in the study, of which 47% (28 out of 60) belonged to female patients. The average age for all participants at the time of scanning was 51.2 years, with female patients having a mean age of 59.5 years and male patients averaging 43.9 years. The scans were predominantly conducted for various clinical reasons: 26 for fractures of the distal radius, 1 for a metacarpal fracture, 10 for scaphoid fractures, 2 for assessing the base-of-thumb OA, 1 for a hamate fracture, 1 for the dislocation of the fourth/fifth metacarpal, 1 for gout, and 18 for general assessments of fractures or dislocations. The right hand was the subject of the scans 77% (46 out of 60) of the time. In terms of the presence of OA at the base of the thumb, 45% (27 out of 60) of the patients showed no evidence, 38.3% (23 out of 60) had stage II OA, 3.3% (2 out of 60) had stage III OA, and 13.3% (8 out of 60) had stage IV OA.

### Demographics of Brown University

The dataset comprised 60 patients, and 50% of the population were female. The average age of the patients was 24.7 years; among female patients, the mean age stood at 24.4 years, while it was 25 years for male patients. Age-related data were unavailable for 20 of the subjects, and this portion was subsequently omitted from the analysis. As for the rationale behind the imaging, all scans were performed solely for research and study objectives. Importantly, all participants were healthy and exhibited no signs of OA at the base of the thumb.

### Demographics of Auckland University

The dataset from Auckland University contained 61 patients, and 52.5% (32/61) of them were female. The mean age of the female patients was 42.8 years, and 40.3 years for male patients. The imaging was done for research purposes, and all patients were healthy without any evidence of base-of-thumb OA.

### User Variably in 3D Slicer–Generated Carpal Bone Volume

The intrauser variability or agreement between blinded repeat measurements of the same trapezium and pisiform bones had an average ICC of 0.86 (confidence interval [CI] 0.75-0.92). This corresponded to “good” reliability between repeated measurements and was statistically significant (*P* < .05).^15^ The interuser similarity or agreement between blinded repeat measurements of the same trapezium and pisiform bones had an average ICC of 0.95 (CI 0.95-0.97). This corresponded to “Excellent” reliability between repeated measurements and was statistically significant (*P* = .00).

### Data Distribution

The datasets from Peninsula Health and Auckland University displayed normal distributions, as indicated by Shapiro-Wilk test results with *P*-values of .153 and .188, respectively. In contrast, the data from Brown University did not follow a normal distribution, as evidenced by a Shapiro-Wilk test *P*-value of .00129. Consequently, the aggregated dataset was analyzed using Spearman’s rank-order correlation, which is appropriate for nonparametric data.

### Spearman Correlation Between Carpal Bone Volume and Age in Males and Females

**Figure 2.**
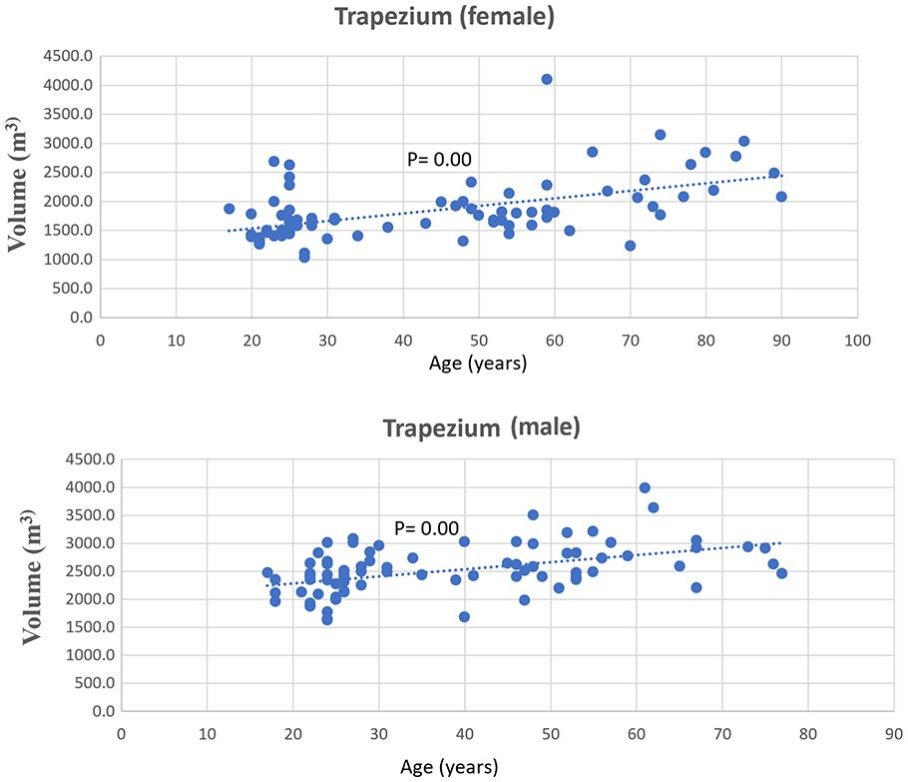
Spearman correlation between trapezium volume and age in males (rho = 0.51) and females (rho = 0.54).

**Figure 3.**
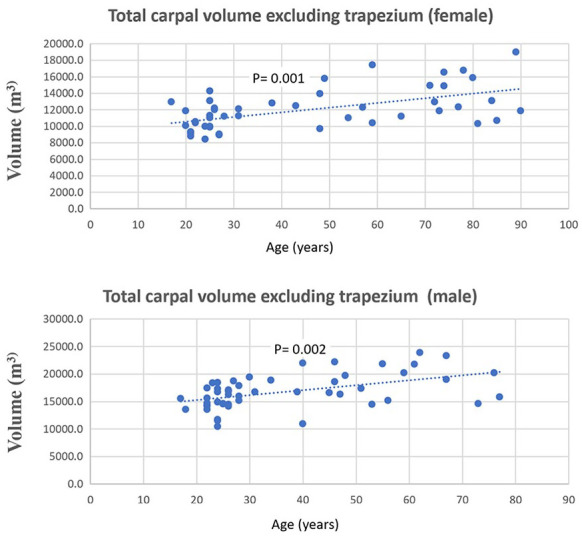
Spearman correlation between total carpal volume excluding the trapezium and age in males (rho = 0.42) and females (rho = 0.45).

A statistically significant positive (*P*-value < .05) Spearman correlation was found between the volume of each carpal bone and age, with rho values ranging from 0.37 to 0.61 (as detailed in Table 1). Figure 2 visually represents this upward trend between the volume of the trapezium and age. Similarly, Figure 3 depicts the trend of increasing total carpal volume (excluding the trapezium) with age, for both males and females.

**Table 1.** The Spearman Correlation Coefficient for Each Carpal Bone and Total Carpus Minus the Trapezium.

Carpal bone	Spearman correlation (rho = 0.37-0.61)	
	Males	Females
Trapezium	0.51	0.54
Scaphoid	0.42	0.45
Lunate	0.45	0.61
Triquetrum	0.37	0.52
Pisiform	0.53	0.52
Trapezoid	0.50	0.49
Capitate	0.52	0.50
Hamate	0.52	0.37
Total carpal volume (excluding trapezium)	0.42	0.45

## Discussion

The current study establishes the changes in carpal bone volume with aging. The results of the study show a positive Spearman correlation between each carpal bone volume and increasing age. This positive correlation may be partially explained by the increasing prevalence of OA with age,^16^ especially OA affecting the base of the thumb, which is notably common among middle-aged (postmenopausal) and older women.^17^ In the context of OA, changes such as the formation of osteophytes can lead to an increased net volume of the trapezium, even when there’s a reduction in trapezial height.

Interestingly, while OA impacts joints like the scapho-trapezial and trapezo-trapezoid, other carpal bones such as the pisiform remain largely unaffected by OA, even in older individuals. In addition, when the total carpal volume was recalculated excluding the trapezium, which could potentially skew the results due to osteophyte formation, the same upward trend in volume relative to age was observed. This observation raises intriguing questions regarding the underlying mechanisms that drive volume changes in carpal bones not directly influenced by OA, prompting further research into factors such as subchondral bone density alterations or adaptational bone remodeling. One plausible explanation could be alterations in subchondral bone density, which plays a critical role in joint health and OA development. Subchondral bone alterations, such as increased bone turnover and density changes, have been implicated in OA and could contribute to the observed volume changes. Another aspect to consider is adaptational bone remodeling. Bones are dynamic structures that remodel in response to mechanical stress and age-related physiological changes. This remodeling process, involving the resorption and formation of bone tissue, could lead to variations in bone volume. In the context of the carpus, which is subject to varying mechanical loads and movements, such remodeling might be particularly relevant. These observations necessitate further research to elucidate the mechanisms driving volume changes in carpal bones, especially those not directly influenced by OA.

Age-related carpal bone volume changes involve various physiological mechanisms and alterations in bone density. Studies have shown that between the ages of 20 and 90 years, there is a decrease in trabecular bone volume/tissue volume in both men and women.^18^ It has been described that smaller carpal bone volumes were predictive of the onset of OA in the wrist.^19^ The term “volume” often refers to a reduction in bone density and the integrity of trabecular bone, rather than a decrease in the physical space occupied by the bone.^19^ This reduction in bone density, characterized by a loss of trabecular structure, contrasts with an actual increase in bone volume due to the formation of osteophytes, which are symptomatic of joint instability and inflammation.^19^ These complex changes in bone composition and structure, involving both loss of density and volumetric increase, highlight the intricate nature of bone remodeling in OA and underscore the importance of precise diagnostic techniques in its management.^19^ While it is possible to isolate and segment osteophytes from imaging data for a more precise assessment of true bone volume changes characteristic of OA progression, this was not the primarily focused area of this study. This relationship between reduced bone volume and OA can be understood better when considering the role of subchondral bone in the pathogenesis of OA.^20^ As the bone volume decreases, the subchondral bone thickens and becomes sclerotic.^20^ This change has been associated with the onset of OA due to altered stress distributions in the joint and increased cartilage loading. Moreover, age-related changes in the ligaments and soft tissues surrounding the carpus further complicate the scenario.^4^ Ligaments can become lax or undergo degenerative changes, leading to altered carpal kinematics and increasing the risk for conditions like carpal instability.^21,22^ Understanding these age-related changes in carpal bone volume holds significant clinical value, particularly for the diagnosis and management of OA and other degenerative musculoskeletal conditions. The data could refine current therapeutic approaches, such as surgical methods and postoperative care tailored for older patients, and could also inform proactive interventions like lifestyle modifications or pharmacological treatments aimed at delaying the onset or progression of OA.

Interestingly, there is evidence to suggest that hand size increases with age, although the underlying mechanism is not fully understood.^23,24^ A prior study found that hand length and width increased significantly with age in both men and women. This augmentation in hand size is largely attributed to alterations in the skeletal architecture of the hand itself.^25^ In addition, age-related shifts in bone strength characteristics have been observed, as evidenced by radiographic densitometry of the second metacarpal bone indicating a risk gradient. Bone changes occurring during normal aging are a universal phenomenon, and bone loss probably does not differ greatly between men and women.^23^ Age-related patterns of trabecular and cortical bone loss differ between sexes and skeletal sites, with trabecular bone volume/tissue volume decreasing in both men and women between the ages of 20 and 90 years.^26^ Overall, age-related increases in hand size are likely due to changes in the bone structure of the hand, which may be related to age-related changes in bone strength phenotypes and trabecular and cortical bone loss.

The biological rationale for an increase in carpal bone size with advancing age remains open to debate. While the current cross-sectional study design revealed a positive correlation, these results could be influenced by individual variability in bone morphology rather than representing a true age-related trend. Tendon insertions, akin to those affecting the distal and middle phalanges, may partly account for the observed changes in specific carpal bones. For example, the pisiform and hamate accommodate insertions from the flexor carpi ulnaris, and the trapezial tuberosity receives a slip from the flexor carpi radialis. However, this mechanism falls short of explaining trends in bones like the capitate or lunate, which do not have tendon insertions. Another possible explanation could be the ongoing physiological bone turnover, a lifelong process that may result in incremental increases in carpal size. The current study, by its cross-sectional nature, only establishes a correlation rather than causation. A more definitive answer could potentially be achieved through a longitudinal design that tracks individual carpal volumes over several years. However, the logistical challenges of such a study are considerable. Our stratification of data by gender, while insightful, also limited the granularity of further stratifications such as defined age groups. Doing so with our existing dataset could have led to insufficient sample sizes in each subgroup, raising the risk of false negatives or undetectable significant differences.

Although our study demonstrates a notable correlation between carpal bone volume and age, it is essential to consider its limitations. Our study did not account for interindividual differences, such as those attributable to ethnicity. Specifically, the dataset from Peninsula Health may have a bias toward a predominantly Western European population, given its location in the Mornington Peninsula of Victoria, Australia, a region with less ethnic diversity than the broader Melbourne area. Future studies may benefit from a more ethnically diverse sample to provide a comprehensive view of age-related changes in carpal bone volumes. In addition, we did not compare the calculated volumes between the two groups of CT scans—those with 0.5-mm voxels and those with 1-mm voxels. Therefore, it remains undetermined if significant differences in volume measurements might exist due to the differing voxel sizes, which could potentially impact the precision of our findings. Furthermore, the study had a low sample size and was of a cross-sectional design, limiting our ability to establish causal relationships between age and changes in carpal bone volume. A longitudinal study, tracking the same individuals over an extended period, would provide more robust evidence for causality. However, conducting such a study may present practical challenges, including logistical and ethical considerations. In addition, the potential impact of other factors like occupation, physical activity, and health conditions on carpal bone volume was not considered, which could introduce confounding variables into the analysis. It is also worth mentioning that our study did not explore the underlying pathophysiological mechanisms that might explain the observed correlation. While we discussed the possible role of OA and other physiological processes, these were not specifically investigated. Consequently, the biological basis for the increase in carpal bone volume with age remains speculative at this stage.

Our study identifies a positive correlation between age and carpal bone volume, including in bones largely unaffected by OA. These findings suggest that factors other than tendon insertions and OA may contribute to age-related changes in carpal bone volume. Nonetheless, the biological mechanisms behind these observations remain unclear, warranting future longitudinal research with a larger sample size.
